# Advanced Ultrasound Imaging in Glioma Surgery: Beyond Gray-Scale B-mode

**DOI:** 10.3389/fonc.2018.00576

**Published:** 2018-12-03

**Authors:** Massimiliano Del Bene, Alessandro Perin, Cecilia Casali, Federico Legnani, Andrea Saladino, Luca Mattei, Ignazio Gaspare Vetrano, Marco Saini, Francesco DiMeco, Francesco Prada

**Affiliations:** ^1^Department of Neurosurgery, Fondazione IRCCS Istituto Neurologico Carlo Besta, Milan, Italy; ^2^Department of Experimental Oncology, IEO, European Institute of Oncology IRCCS, Milan, Italy; ^3^Department of Pathophysiology and Transplantation, University of Milan, Milan, Italy; ^4^Department of Neurological Surgery, Johns Hopkins Medical School, Baltimore, MD, United States; ^5^Department of Neurological Surgery, University of Virginia, Charlottesville, VA, United States; ^6^Focused Ultrasound Foundation, Charlottesville, VA, United States

**Keywords:** Glioma, intra-operative ultrasound, contrast enhanced ultrasound, Doppler, B-mode, elastography, fusion imaging, navigated ultrasound

## Abstract

**Introduction:** Glioma surgery is aimed at obtaining maximal safe tumor resection while preserving or improving patient's neurological status. For this reason, there is growing interest for intra-operative imaging in neuro-oncological surgery. Intra-operative ultrasound (ioUS) provides the surgeon with real-time, anatomical and functional information. Despite this, in neurosurgery ioUS mainly relies only on gray-scale brightness mode (B-mode). Many other ultrasound imaging modalities, such as Fusion Imaging with pre-operative acquired magnetic resonance imaging (MRI), Doppler modes, Contrast Enhanced Ultrasound (CEUS), and elastosonography have been developed and have been extensively used in other organs. Although these modalities offer valuable real-time intra-operative information, so far their usage during neurosurgical procedures is still limited.

**Purpose:** To present an US-based multimodal approach for image-guidance in glioma surgery, highlighting the different features of advanced US modalities: fusion imaging with pre-operative acquired MRI for Virtual Navigation, B-mode, Doppler (power-, color-, spectral-), CEUS, and elastosonography.

**Methods:** We describe, in a step-by-step fashion, the applications of the most relevant advanced US modalities during different stages of surgery and their implications for surgical decision-making. Each US modality is illustrated from a technical standpoint and its application during glioma surgery is discussed.

**Results:** B-mode offers dynamic morphological information, which can be further implemented with fusion imaging to improve image understanding and orientation. Doppler imaging permits to evaluate anatomy and function of the vascular tree. CEUS allows to perform a real-time angiosonography, providing valuable information in regards of parenchyma and tumor vascularization and perfusion. This facilitates tumor detection and surgical strategy, also allowing to characterize tumor grade and to identify residual tumor. Elastosonography is a promising tool able to better define tumor margins, parenchymal infiltration, tumor consistency and permitting differentiation of high grade and low grade lesions.

**Conclusions:** Multimodal ioUS represents a valuable tool for glioma surgery being highly informative, rapid, repeatable, and real-time. It is able to differentiate low grade from high grade tumors and to provide the surgeon with relevant information for surgical decision-making. ioUS could be integrated with other intra-operative imaging and functional approaches in a synergistic manner to offer the best image guidance for each patient.

## Introduction

Extent of resection (EOR) together with brain function sparing represent the most critical aspects of glioma surgery. A growing body of literature and evidences firmly supports gross total removal (GTR), instead of subtotal (STR) or biopsy (1, 2), defining GTR as the complete resection of contrast-enhancing regions in high grade glioma (HGG) on T1 weighted Gd-enhanced magnetic resonance imaging (MRI), and of hyper-intense areas on T2/FLAIR MRI in non-enhancing low grade glioma (LGG).

In diffuse LGG, GTR is able to improve progression free survival (PFS), overall survival (OS) and the time needed for malignant transformation (1–5). In HGG, GTR is nowadays considered the first phase of the standard multimodal therapeutic approach in order to extend PFS and OS (1, 2).

Surgeon's perception of gross total removal in glioma surgery is commonly inaccurate (6): portions of intra-axial tumor may resemble healthy brain parenchyma thus leading to sub-optimal resection and subsequently influencing patient's prognosis.

With these premises a growing interest for intra-operative imaging has led to the development of new technologies to localize tumors in order to ultimately help surgeons achieving GTR (7).

Numerous intra-operative approaches have been proposed: computed tomography (ioCT), magnetic resonance imaging (ioMRI), ultrasound (ioUS), fluorescence guided surgery (FGS) [e.g., 5-ALA, fluorescein, second window idocyanine green (ICG)] and other experimental techniques (e.g., optical coherence tomography and Raman spectroscopy) (7–14).

Among all these techniques ioUS is still one of the most employed, studied and developed, despite being the most dated since its first report was in 1978 with Reid (15).

US application in brain is especially favored by cerebral mechanical properties which allow an excellent US propagation and by the absence of superficial layers such as skin and subcutaneous connective which can distort US waves (16).

The main value of ioUS is the possibility to study the surgical scenario in real-time, every time it is needed, without the interruption of surgical work flow and, in specific condition, permitting to operate under direct guidance (17).

Continuous research and development led to US probes and scanners able to provide images with superb temporal and spatial resolution, comparable, or even superior to volumetric MRI (18).

Numerous studies have also investigated the diagnostic properties of ioUS in terms of sensitivity, specificity and ability to increase EOR and subsequently PFS and OS (9, 19–22).

In general ioUS demonstrated to own high diagnostic value in glioma surgery, in particular in low grade lesions, allowing to maximize the extent of resection and consequently to improve prognosis and quality of life of patients (9, 19–25).

It has to be said that in most of the cases ioUS application is limited to standard brightness mode (B-mode) with or without co-registration to pre-operative MRI. B-mode alone, being an anatomical representation of the echo wave for each point in the space, is a truncated application for ioUS. Indeed one pivotal adjunct of this technology is the possibility to implement different modalities to broaden the amount of different information.

The aim of our work is to review and describe different ioUS modalities in glioma surgery underlying the potential implications of standard b-mode and other advanced techniques such as fusion imaging, Doppler (power-, color-, spectral-), CEUS, and elastosography.

## Intraoperative Ultrasound in Glioma Surgery

### US Equipment

US scanner should include the predisposition for different US modalities, a tracking system, the possibility to support different probes, the option to modify imaging presets through a complete access to all the US parameters. In general a specific designation for neurosurgery application is not required, as in most of cases a last generation general radiology US scanner with different presets is sufficient (10).

The scanner should provide a tracking system to allow fusion imaging with pre-operative MRI, to correct brain shift, and also to acquire a 3D US scan to obtain an updated neuronavigation volume.

The system should be equipped with different probes: a linear multifrequency (3–11 MHz) probe for deep-seated lesion, an high frequency (10–22 MHz) for small superficial lesions and a mini-convex to study the surgical field from inside the surgical cavity, overcoming the limitations of surgical artifacts in the final stages of tumor resection (10, 26).

Another issue is represented by imaging presets: in most US scanner designed for neurosurgery, imaging preset is standardized in order to provide highly contrasted image with few modifiable parameters. US is a demanding technique, with a steep learning curve and high operator dependency, but the only way to obtain the maximum in every situation is to became accustomed to this imaging modality and consequently being able to master the settings accordingly to the scenario (27).

The typical workflow at our Institution comprise the use of a last generation US scanner (MyLab, Esaote, Italy) with an integrated magnetic tracking system allowing for virtual navigation (MedCom GmbH, Germany). Typically the patient is registered in the 3D frame to the pre-operative MRI volume, permitting fusion imaging between ioUS and MRI and also to navigate with a pointer (as with a standard navigation system), to plan the surgical strategy and designing craniotomy site and shape (28). After bone flap removal the probes (usually a linear multifrequency and a mini-convex) are wrapped in plastic sterile sheath with coupling sterile US gel (Civco, USA) and a first direct trans-dural insonation is performed. The field is continuously irrigated with saline solution to allow US coupling and to improve imaging, reducing air or blood clots between probe and brain/dura. In every case the first US modality applied is B-mode, usually followed by Doppler, CEUS, elastography, depending on which information is necessary to achieve.

Indeed B-mode provides anatomical information requisite in order to understand and exploit the other modalities. Furthermore, B-mode permits to correct the brain-shift and brain deformation that naturally take place as resection advances (29, 30). Multiple US scans are performed throughout the whole surgery. Once tumor resection is completed, the final multimodal scan is conducted to evaluate potential hidden residual tumor and hypothetical tissue and vessels damages.

### B-mode ioUS

**Figure 1:**
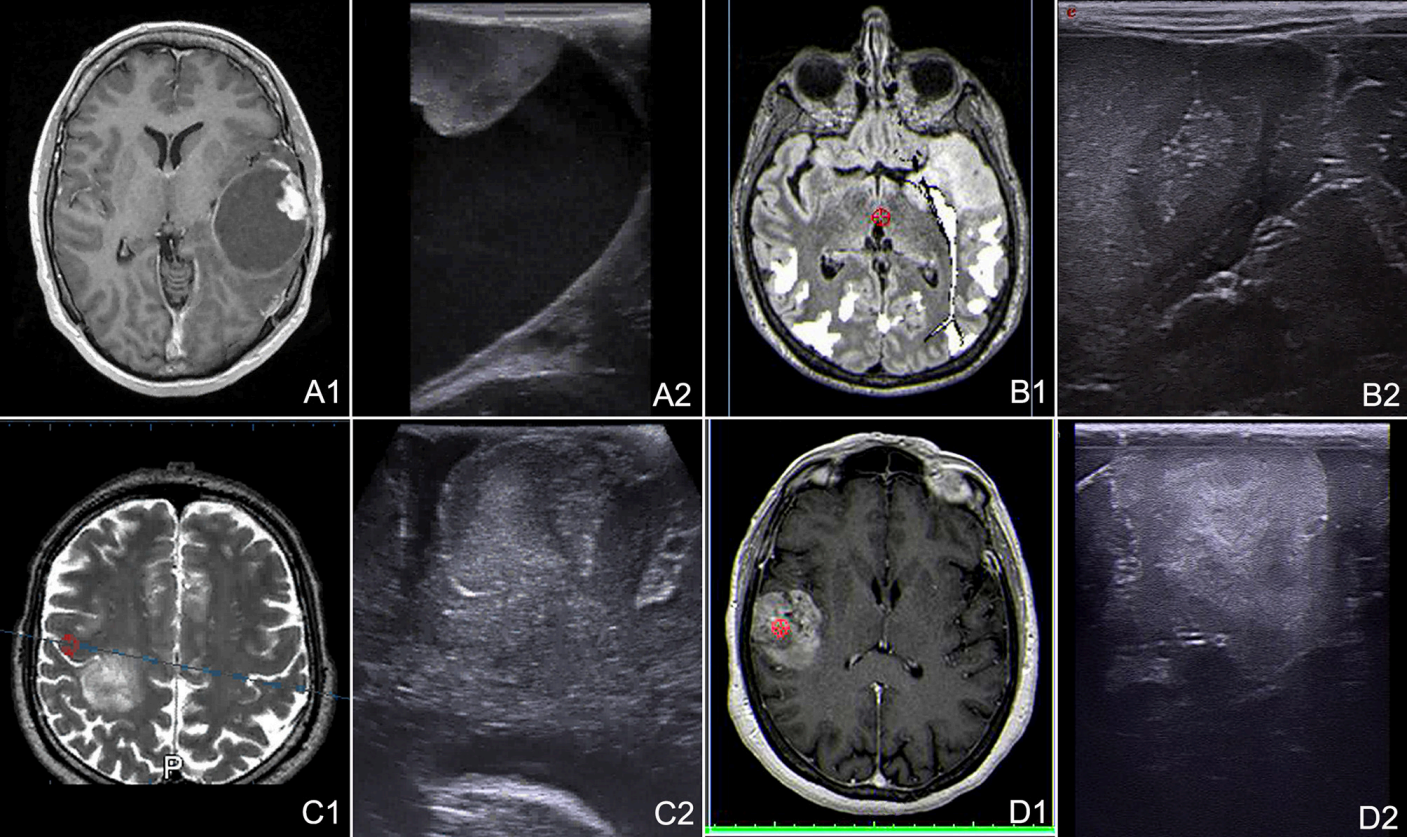
B-mode representation of different glioma grades. **(A)** Left temporal pilocytic astrocytoma. **(B)** Left temporal diffuse astrocytoma. **(C)** Right parietal anaplastic astrocytoma. **(D)** Right temporo-parietal glioblastoma. (1) pre-operative volumetric MRI. (2) Intra-operative trans-dural US scan. Note the different lesion appearances and in particular the different degree of margins definition.

**Figure 2:**
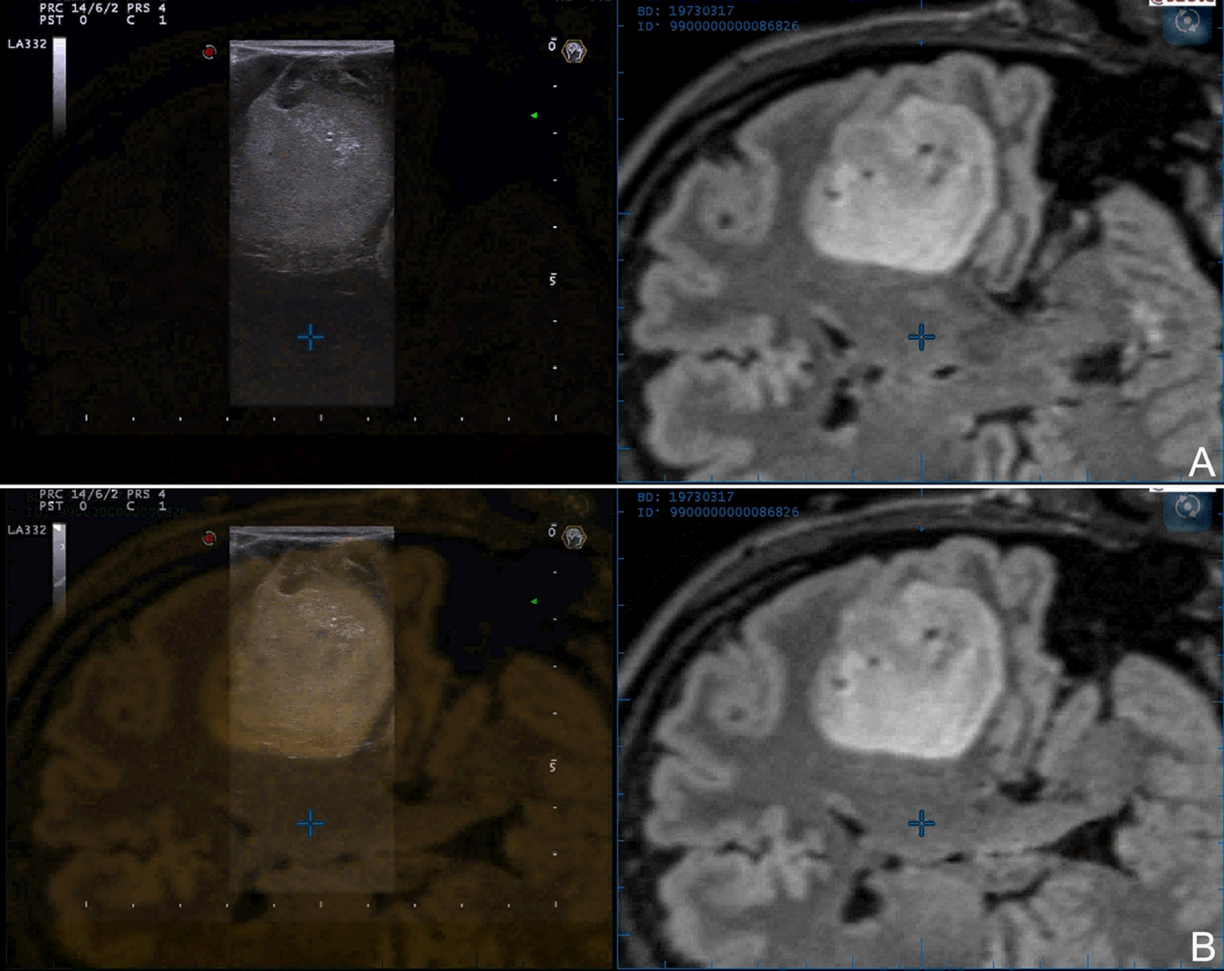
Navigated intra-operative B-mode US in a case of left temporo-insular low-grade glioma. Two different configurations of navigated ioUS are displayed: **(A)** side-by-side or **(B)** superimposition. The continuous comparison between the two modalities aid in orientation and understanding of ioUS images.

**Figure 3:**
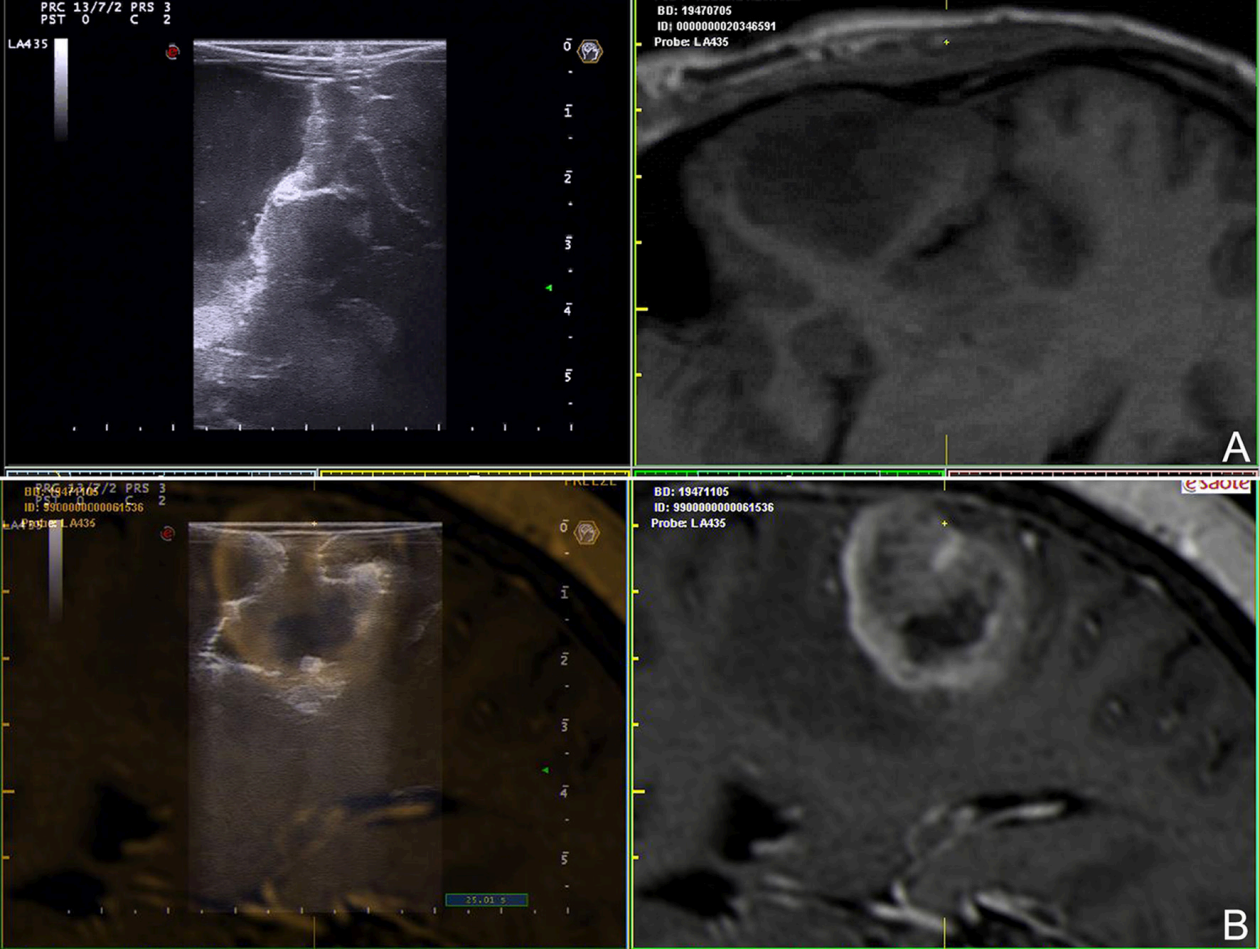
Navigated intra-operative B-mode US in the final stages of surgery. **(A)** Left temporal anaplastic astrocytoma. **(B)** Right fronto-temporal glioblastoma. The comparison between ioUS and corresponding pre-operative MRI aid in identifying residual tumor and understanding ioUS semiotics: **(A)** side-by-side view, **(B)** superimposition.

B-mode is the most simple and diffuse modality of US imaging, such that in some cases “US” and “B-mode” are erroneously used as synonyms. Literally, B-mode stands for Brightness mode, a two-dimensional US imaging modality formed by bright dots, which represent the amplitude of each reflected eco-wave in a specific point in the space. B-mode permits to visualize and characterize anatomical structures relying on their capacity to reflect, refract, absorb or transmit US beam (31, 32) (Figures 1–3). The brightness of a structure of interest (e.g., tumor) is evaluated in comparison to surrounding healthy tissue and consequently a structure can be hyperechoic, hypoechoic, or isoechoic. It has to be said that neurosurgical US semiotics is demanding especially for an un-experienced user. In general, structures defined as hyperechoic are: ependyma, choroid plexus, arachnoid interfaces, dural structures, skull, most tumors and their margins. Cisterns, ventricles, cerebro-spinal fluid, and some tumors tend to be hypoechogenic. Gray matter, white matter (typically gray matter is brighter than white matter) and some tumors appear as isoechoic (33, 34) (Figures 1–3).

Glioma appearance in ioUS B-mode is dependent on lesion grading and consequently biological behavior (Figure 1). HGG own an explosive growth, with high proliferation and areas of cysts, bleedings, necrosis, high-cells-density, and invasive zones. All these features lead to an heterogeneous representation in which is possible to identify the different areas of the lesion. In general, it is possible to say that HGG appear heterogeneously echogenic with hyperechoic margins and iso-hypoechoic central necrotic areas. In general, the margins are more identifiable than in LGG even if is difficult to differentiate between tumor boundaries and peri-lesional edema (10, 23, 33, 35–39) (Figure 1). On the other hand, LGG appear slightly hyperechoic if compared to healthy brain, with homogeneous aspect and blurred margins particularly where they merge with healthy white matter (Figure 1). In most of cases B-mode imaging overlap pre-operative FLAIR MRI scan in LGG (Figure 2). The main difficulty in these tumors is to identify the margins/areas of invasion from peri-lesional edema (23, 33, 35–38).

Numerous studies have reported on the applications of B-mode in oncological neurosurgery and in particular in glioma resection (9, 10, 19–26). Several reviews and meta-analyses have addressed the value of ioUS in glioma surgery even if it is still not available a randomized controlled trial as pointed out in the last version of Cochrane review on intra-operative imaging technologies to enhance EOR in glioma (7).

In his meta-analysis Guangying Zhang found that B-mode provides an high sensitivity and specificity (0.75 and 0.88) in identifying tumor residual in glioma surgery operation, especially in LGG. In their study the Authors also confirmed the difficulty in distinguishing edema from infiltrating zone in particular for HGG and the ability of B-mode to display tumor presence also in areas with preserved blood brain barrier (21).

Bodil Karoline Ravn Munkvold performed a review on the diagnostic properties of ioUS in glioma surgery and analyzed factors influencing EOR. He found an overall specificity of 85% while sensitivity was 46% even if the residual tumor was small (median 1.05 ml) in cases with false-negative ioUS. Specificity was higher in LGG than in HGG (94 and 77%) and lowest in patients who undergone previous radiotherapy. The Authors conclude their analysis stating that ioUS specificity is high while the sensitivity for small residue is lower than post-operative MRI (22).

Syed Mahboob conducted a meta-analysis of the existing literature on the application of ioUS B-mode in glioma surgery. He analyzed 739 cases of LGG an HGG glioma operated under ioUS B-mode guidance in which gross total resection was achieved in 77% of patients (HGG 71.9% and LGG 78.1%). The Authors also examined, through a multivariate analysis, the factors implied in GTR finding that ioUS image quality is one of those and in turn it is influenced by previous surgery and radiotherapy. The Authors conclude that ioUS is able to improve EOR, especially in conjunction with other technologies to enhance anatomic orientation (9).

Jia Wang in his study investigated the role of ioUS in improving the survival time of patients who underwent resection of cerebral gliomas. He compared the survival rate at 6 months, 1-year, and 2-year in LGG and HGG patients operated with and without ioUS. He observed that in those patients in which ioUS was used survival rate at 1 and 2 year were significantly better than the survival rates of the controls (19). Their results were confirmed by Saether et al. in another retrospective study (40).

In our experience B-mode is extremely helpful in each phase of surgery. Before dural opening, it permits to find the lesion, to study its extension and if necessary to modify the craniotomy accordingly (Figure 1). Once dura is opened, brain shift take place, and consequently anatomy could be importantly modified. In this context B-mode allows to find the lesion, neighbor anatomical landmarks and vital structures (Figure 1). Furthermore, B-mode provides information on which gyrus is infiltrated and which is spared thus allowing to tailor the corticectomy according to lesion extension. Notably, being ioUS real-time, in case of discordance between US and neuronavigation, B-mode provide the most reliable and updated information (28). If the lesion is deep-seated, B-mode permits to plan the surgical corridor and if necessary to select the appropriate sulcus for a trans-sulcal approach. During surgical removal B-mode is repeated several times, to understand the dynamic surgical anatomy (e.g., inform on the distance to ventricles or vital structures) and to guide other ioUS modalities such as Doppler, CEUS, and elastography. In our experience repeated ioUS scan permits to be more confident in surgical resection and at the same time to be more efficient and safer.

At the end of surgical resection, a last scan is performed to identify potential residual tumor (10) (Figure 3). In case of doubt, more advanced imaging such as CEUS can be employed.

It has to be said that as surgical resection advances the ioUS image quality decrease (Figures 1, 3). The sensitivity, specificity and derived values (positive predictive value, negative predictive value) are optimal before surgical resection and deteriorate as surgery proceeds (41). This is mainly due to surgical induced artifacts and edema and as a consequence several approaches have been proposed to overcome this limitation.

Navigating the US probe it is possible to compare the location of an hyperechoic area in ioUS with the tumor extension on pre-operative MRI; if the suspected tumor is outside the tumor area in the pre-operative imaging it is likely to be an artifact (25, 28, 37, 42–47) (Figures 2, 3). Notably this approach can be only suggestive because even correcting the brain shift it is still not possible to correct brain deformation.

Selbekk et al. in 2013 proposed the use of a special coupling fluid to fill the surgical cavity. The hypothesis is that surgical artifacts are related to different acoustic coefficients of saline water and brain parenchyma thus inducing a bright artifact in surgical cavity wall. In order to overcome this limitation the Authors proposed a fluid with the same attenuation coefficient of human brain (48). Even if really interesting this approach is still experimental and is far from being routinely used in clinical practice.

Šteno et al. identified the cause of brightness artifacts in the column of water in the surgical cavity and as a consequence in the distance between probe and surgical bed (26, 36, 43). They proposed the application of miniature high frequency probes to scan the border of the surgical cavity from inside. The main limitation of this approach is the physical characteristics of these probes that are limited in field of view, lateral resolution, and US penetration.

In our experience the most reliable solution to overcome surgical induced artifacts and to discriminate between them, residual tumor and tumor induced edema is CEUS and we will analyze this application in the specific section below.

Tumor recognition in different phases of surgical resection is only one of the limitations of B-mode.

ioUS has to pay the steep learning curve and operator dependency mainly related to orientation and semiotics interpretation. Usually, neurosurgeons are accustomed to standard orthogonal planes (axial, coronal, and sagittal) while ioUS provides oblique planes dependent on probe location and orientation. In our experience fusion imaging with pre-operative MRI is extremely helpful to overcome this issue especially in case of an un-experienced operator (Figures 2, 3). The continuous comparison with a familiar imaging allows to understand the orientation and the specific US semiotics, which is dynamic and influenced by surgical resection (10, 25, 28, 42–44, 46, 49). Another solution is 3D ioUS which allows to scan the surgical field obtaining a 3D volume in which is possible to navigate through a pointer in the standard planes (axial, coronal and sagittal) (10, 24, 25, 40–44). In our opinion this represent a really useful solution that can facilitates ioUS understanding and permits to navigate in an update 3D volume. At the same time, US scan, being a volumetric acquisition, bears less information than 2D US. Indeed 3D US does not allow to take advantage from the proprioceptive feedback and eye-hand coordination to reconstruct a real-time mental representation of the surgical field, as it is performed by sonologists in other corporeal regions to explore different relationships between structures (26).

### Navigated ioUS

Cerbral US is not a familiar imaging for neurosurgeons. This is due to the impossibility to use US in pre- and post-surgical phases, whereas the opposite is true for diagnostic imaging such as MRI and CT, which own specific semiotics and orientation in three orthogonal planes. Furthermore, ioUS is peculiar for several reasons. Image orientation depends on the plane of insonation and consequently on probe orientation and position (Figures 1–3). Semiotics is specific and dynamic among the different phases of surgical resection. US does not permit to study intracranial space before bone removal and to plan craniotomy because of bone shielding (28) (Figures 1–3).

**Figure 4:**
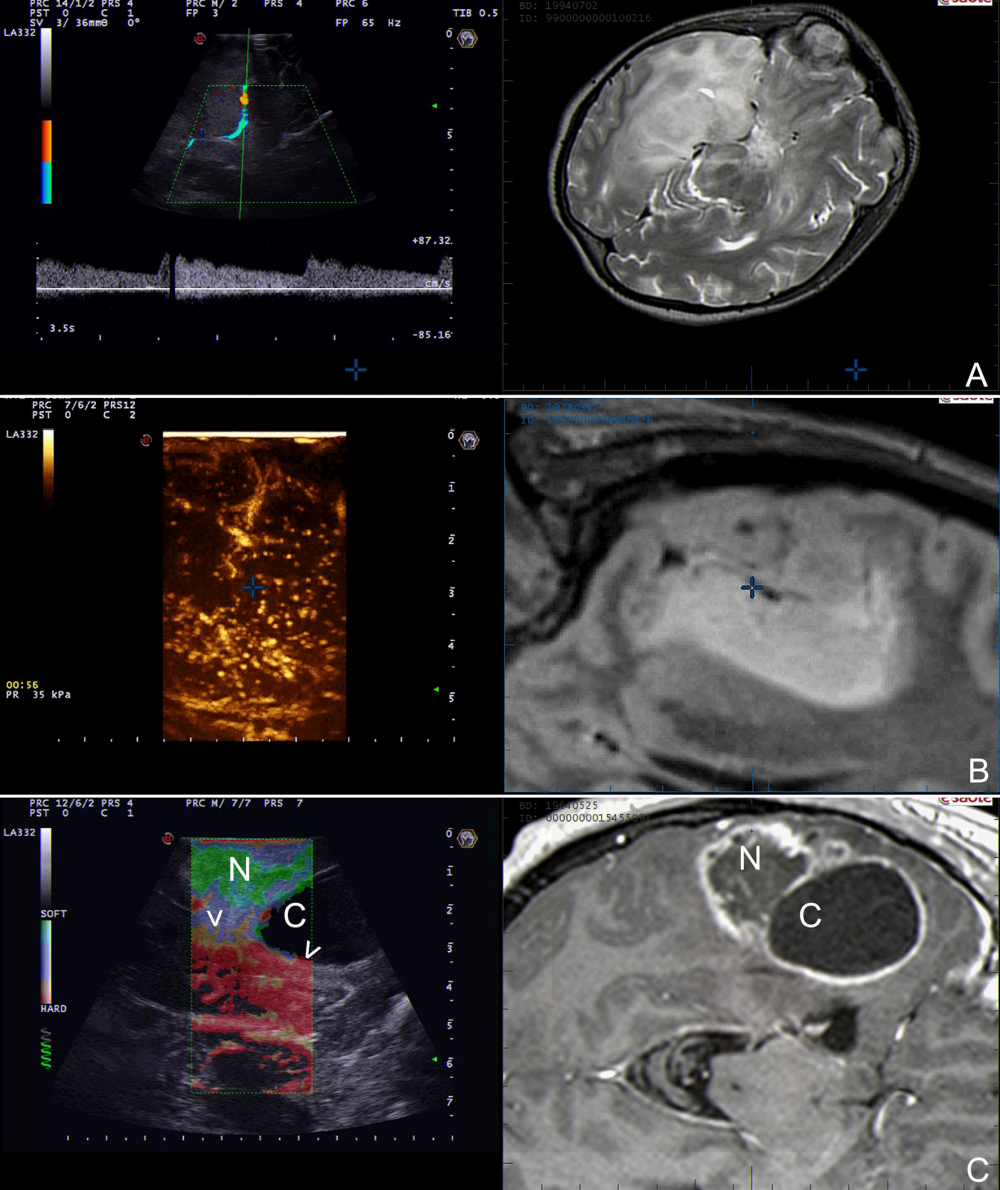
Navigated ioUS (advanced modalities). **(A)** Color and spectral Doppler in a case of left temporo-insular anaplastic oligodendroglioma. **(B)** Contrast enhanced ultrasound in a case of left insular anaplastic astrocytoma. **(C)** Strain elastography in a case of left temporal glioblastoma; note the differences between the necrotic and the cystic areas and the interface with surrounding brain. Exploiting the continuous comparison between ioUS an pre-operative MRI is possible to understand US images and to infer about US and MRI correspondences. Legends are as follow: arrow heads: interface between tumor and brain; N: necrotic part of the tumor; C: cystic part of the tumor.

Fusion imaging permits to co-register ioUS and pre-operative MRI for a continuous comparison of the two imaging modalities, enhancing US understanding and orientation. MRI provides known anatomical details and superimposing or visualizing side-by-side the modalities permits to interpret US orientation, semiotics and to understand its changes during time (Figures 2–4). Furthermore, different MRI datasets can be uploaded such as functional MRI, DTI, perfusion MRI, positron emission tomography in order to understand the location of vital structures, white matter tracts, or more aggressive areas in relation to tumor and real-time surgical situation (24, 25, 28, 41–44, 46). Brain shift can also be corrected relying on real-time US, updating neuronavigation for the most part of surgery while standard neuronavigation can be used to plan the surgical approach (28, 41, 43, 44, 46, 49, 50). Some groups have also demonstrated the possibility to correct brain shift and brain deformation in an automatic fashion through a software analysis taking into account landmarks position in ioUS and deforming MRI images accordingly (50–53).

Numerous Authors have demonstrated the clinical utility of navigated ioUS in glioma surgery allowing to maximize extent of resection and improving patients outcome (10, 19, 25, 40).

In our experience fusion imaging has demonstrated to be a reliable, accurate and useful technique especially for novice US users but also in complex cases and in experimental settings to compare or validate different US modalities in relation to MRI (Figure 4).

### Doppler ioUS

**Figure 5:**
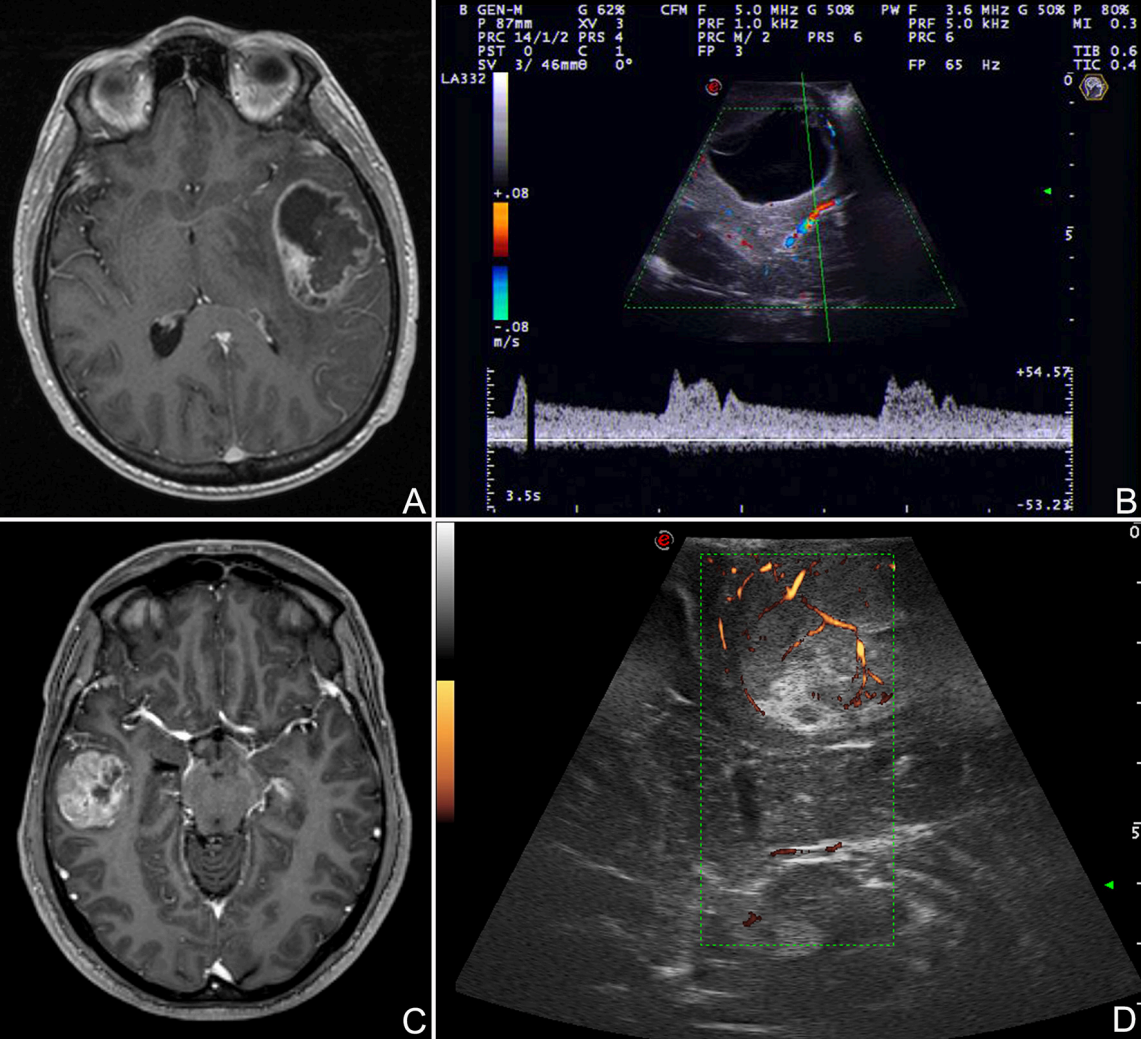
Doppler modalities. **(A,B)** Color Doppler and spectral doppler in a case of left temporal glioblastoma. **(C,D)** Power Doppler in a case of right temporal glioblastoma. Color Doppler informs on presence of flow, its direction and velocity through the setting of a region of interest. Spectral Doppler allows for a systematic analysis of flow-velocity over time thus permitting to characterize vessels nature. Power Doppler provides information on amplitude of flow depicting the number of scattering molecules (mainly erythrocytes).

Doppler US differs from standard B-mode not providing strictly anatomical but rather functional information. It relies on the Doppler effect. When a mechanical wave is reflected by a moving object this generates modifications of frequency and wavelength of echo-waves that can be studied allowing to infer information in regard of vessels blood flow (31). In routine practice, different sub-modalities of doppler are available, depending on which analysis is conducted it is possible to characterize blood flow in specific features (Figure 5). Color Doppler studies the presence of flow, its direction and velocity through the setting of a region of interest (ROI) (Figure 5). US scanners provides these information as color scale superimposed to standard B-mode (31, 54). Power Doppler is more sensitive to the amplitude of flow rather than direction and velocity (Figure 5). The image in encoded in a color scale depicting the total amount of Doppler signal, which in turn is dependent on the number of scattering molecules (in case of blood vessels mainly erythrocytes). This technique is extremely sensitive also to slow flow, typical of capillary district, thus allowing, in some circumstances, to visualize also flow in sub-millimetric vessels (10, 31, 39, 54, 55) (Figure 5). Spectral Doppler requires identification of vessel of interest in B-mode through the setting of a proper ROI and indicating the vessel orientation. This analysis provides a detailed flow-velocity over time graph allowing to characterize vessels nature (e.g., artery, vein) and modification of flow during surgery (31, 54, 55) (Figure 5).

Each of these modalities has its specific indications and drawbacks. Color Doppler is informative in regard of vessels location and flow direction/velocity but at the same time it provides low spatial and temporal resolution and is severely limited by angle of insonation (Figure 5). Power Doppler is less dependent on angle of insonation and provides higher spatial resolution permitting to study also sub-millimetric vessels (Figure 5). On the other hand it is not informative on velocity and direction and suffers from low temporal resolution related to Doppler signal analysis. Lastly, spectral Doppler produces analyses with high temporal resolution allowing to characterize flow velocity pattern and changes in great detail while the drawbacks are the need to set a ROI, the absence of anatomical information and the important influence of angle of insonation (31, 54) (Figure 5).

In glioma surgery, probably, vessels study has less relevance if compared to extra-axial tumor such as skull base meningiomas. In any case, in specific condition this could be very helpful in orienting in the surgical field and in preventing post-operative deficits. Doppler imaging can aid in planning the surgical corridor and dura opening in case of medially located glioma (e.g., midline) according to bridging veins and sinus location (55). In case of deep seated gliomas surfacing on the basal cortex, Doppler allows to identify vessels position and to avoid undesirable damages (e.g., pericallosal arteries or middle cerebral artery branches (10, 49, 55). In specific conditions a vessel can represent a natural landmark for tumor margins location; if this situation is identified on pre-operative MRI, intra-operative Doppler could inform on vessel position thus aiding in achieving GTR avoiding unsafe excesses in eloquent areas (39). In this regard, Steno et al. reported on the feasibility to visualize lenticolo-striatal arteries in insular low grade glioma surgery. The Authors were able to visualize and preserve these small perforators thanks to last generation power Doppler (39).

In our experience Doppler imaging own several indications in glioma surgery, such as vessels flow characterization with spectral Doppler and repeated power Doppler scans in approaching vital structures (e.g., Sylvian vessels). At the same time, we are convinced that in most of the aforementioned applications contrast enhanced ultrasound is more informative and in our routine practice has replaced Doppler imaging.

### Contrast Enhanced ioUS

For other imaging methods such as CT and MRI the use of contrast media is almost mandatory while it is less recognized for ioUS.

The use of CEUS during neuro-oncological procedures has been recently included in the guidelines from the European Federation of the Societies for Ultrasound in Medicine and Biology (EFSUMB), representing a paradigm shift for the use of US in neurosurgery (56).

**Figure 6:**
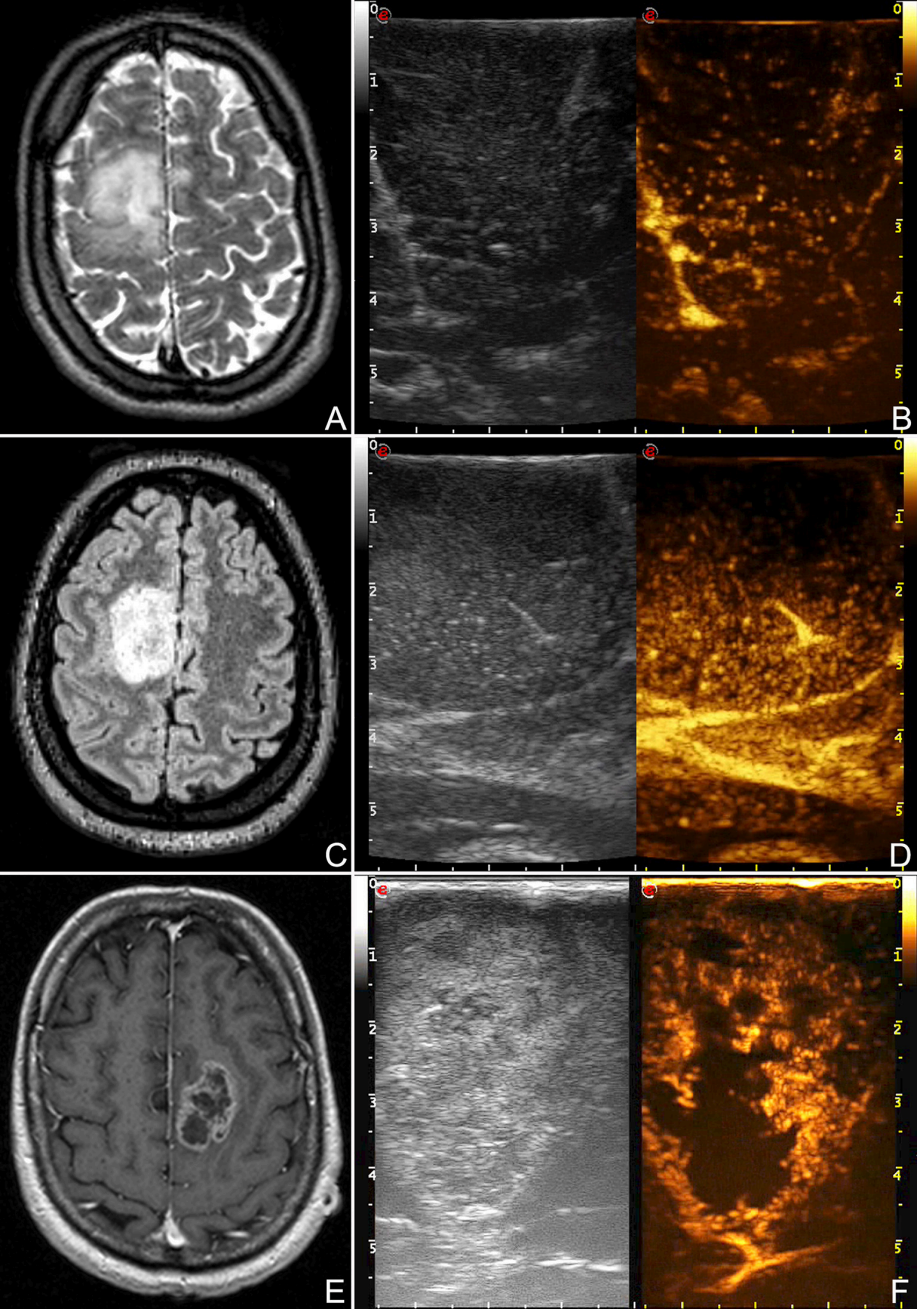
CEUS representation of different glioma grades. **(A,B)** Right frontal low-grade glioma, **(C,D)** right frontal anaplastic astrocytoma, **(E,F)** left frontal glioblastoma. These images demonstrate the different degree and pattern of contrast enhancement among different glioma grades.

**Figure 7:**
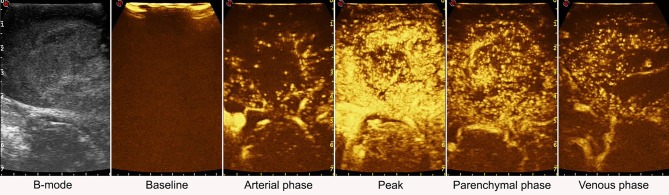
Time-frame of contrast enhancement in a case of right temporal glioblastoma. GBM have a rapid arterial and venous phase. MBs transit is chaotic and the peak is extremely intense. The major arterial supplies and draining veins are clearly visible. Contrast enhancement pattern is irregular and heterogeneous CE with both nodular high-enhanced and hypoperfused areas. Tumor borders are better defined than in B-mode.

**Figure 8:**
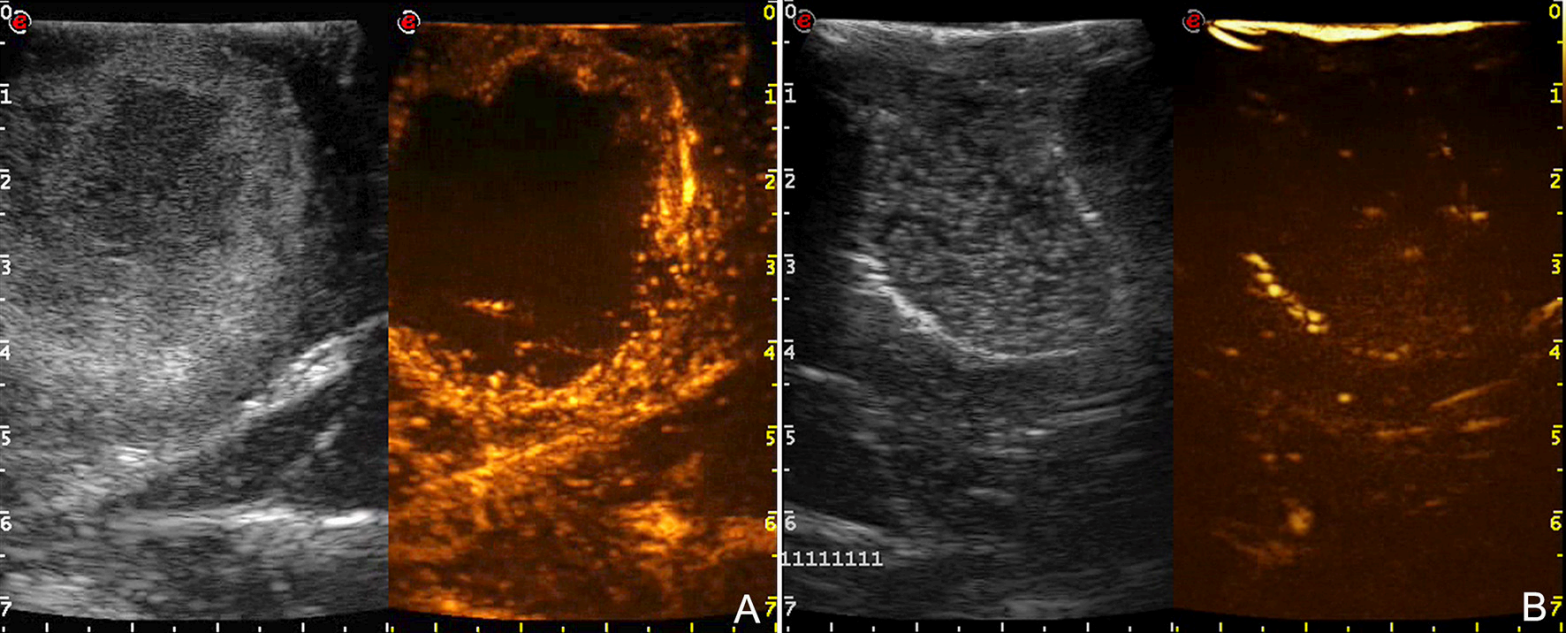
Comparison between pre- **(A)** and post-resection **(B)** CEUS scans in a case of right temporo-parietal glioblastoma. After tumor removal, B-mode became difficult to understand because of surgical-induced artifacts whereas CEUS clearly demonstrates the presence of potential residual tumor.

Contrast enhanced ultrasound (CEUS) is an US modality which exploits a contrast agent (UCA) and a specific algorithm to study the cerebral vasculature down to the capillary bed (Figures 4, 6–8). Nowadays, second-generation UCA are suspensions for venous administration of gas filled micro-bubbles stabilized by a phospholipid shell (MB) allowing for a dynamic and continuous imaging (10, 35, 55–59) (Figure 7). US scanner must be set to low-mechanical index acoustic power in order to induce MB oscillation (minimizing disruption) and consequently to produce a non-linear harmonic echo. Exploiting this feature, CEUS algorithm suppresses the linear US echo from tissue and display only the non-linear harmonic echo of MB thus producing a specific representation of MB distribution (10, 56, 59–62) (Figures 4, 6–8).

Furthermore, MBs, being micron-sized, are not able to extravasate from vessels and behave as a purely intravascular contrast agent, allowing to study all districts of the vascular tree: arterial, venous, and capillary (10, 55–59) (Figures 4, 6–8). CEUS is a dynamic modality which permits to visualize tumors by virtue of degree of vascularization, sharing features with other organs with a terminal circulation such as the kidney (Figures 4, 6–8). It is possible to identify four phase of contrast enhancement (CE): arterial phase, peak of CE, parenchymal phase and venous phase (58, 59) (Figure 7). These phases are dependent on tumor vascularization and perfusion pattern and consequently are extremely informative on tumor biology. Our group has extensively studied CEUS application in neurosurgery with a special attention to glioma surgery (35, 37, 45, 55, 57, 58, 63–69). In our experience CEUS demonstrated to be able to (1) highlight tumors and their phases compared to brain parenchyma (35, 57, 69) (Figures 4, 6–8), (2) characterize glioma grade (35) (Figure 6), (3) inform on vascularization and degree of perfusion (57, 69) (Figures 4, 6–8), (4) show vascular rearrangement that take place with tumor removal (37, 55, 69) (Figure 8), (5) highlight residual tumor (37) (Figure 8), (6) aid surgical decision making through serial imaging assessment of surgical anatomy (10) (Figures 4, 6–8).

GBM usually demonstrate a rapid contrast enhancement (CE) with an impetuous arterial phase (2–3 s), a prompt CE peak (3–5 s) followed by a short parenchymal phase and rapid venous phase (5–10 s). It is almost always possible to identify several feeders, which give a centripetal chaotic transit of MB. Venous phase highlights a diffuse drainage system with multiple medullary veins directed toward ventricles. After tumor removal, we observed with CEUS that medullary veins disappear and in some cases it is possible to see arterialized veins to change flow direction after resection (37, 57, 69). Characteristically it is possible to identify two CE patterns in GBM: (1) heterogeneous with nodular high CE spots interspersed by low-CE areas of necrosis and (2) peripheral rim CE surrounding a central core of necrosis without CE. In all cases GBM show a clearly demarcated border after UCA administration due to the different vascularization of tumor and healthy brain parenchyma (35, 57, 69) (Figures 4, 6–8). We also demonstrated that CEUS is able to highlight the same tumor volume of pre-operative MRI with the same CE pattern thus permitting to visualize residual tumor among all surgical phases (37, 69) (Figure 6).

Anaplastic astrocytoma (AA) demonstrated a slower UCA dynamics with longer phases and CE duration. Even in AA, arterial feeders and venous drainage are visible in most of cases but in general less defined than in GBM. CE pattern is usually diffuse with in some cases few scattered areas of higher CE mixed with small hypoperfused areas. Tumor borders are visible but less sharply than in GBM (35, 57) (Figure 6).

LGG are characterized by two behaviors. Astrocytomas tend to resemble AA CE but with phases even slower. Arterial feeders are usually not identifiable, MB transit is organized and regular while venous drainage is diffuse through numerous capillaries and consequently not discernible. CE is diffuse with dotted appearance, only slightly higher than surrounding parenchyma and with blurred margins (35, 57) (Figure 6). On the other hand, oligodendroglioma CE has a tendency to be more rapid than in astrocytoma, owing faster arterial and venous phases which in any case are slower than in AA. CE pattern in homogeneous with sporadic intralesional cysts and calcification. Margins are better defined than in astrocytoma (35, 57).

Lastly, CEUS permits to identify neighbor vascular structures (both arterial and venous) allowing to follow their localization even at the end of the surgery when resection is on the margins and brain shift has made navigation inaccurate thus assuring a safer dissection (10, 55) (Figures 4, 6, 7).

### Intra-Operative Elastosonography

**Figure 9:**
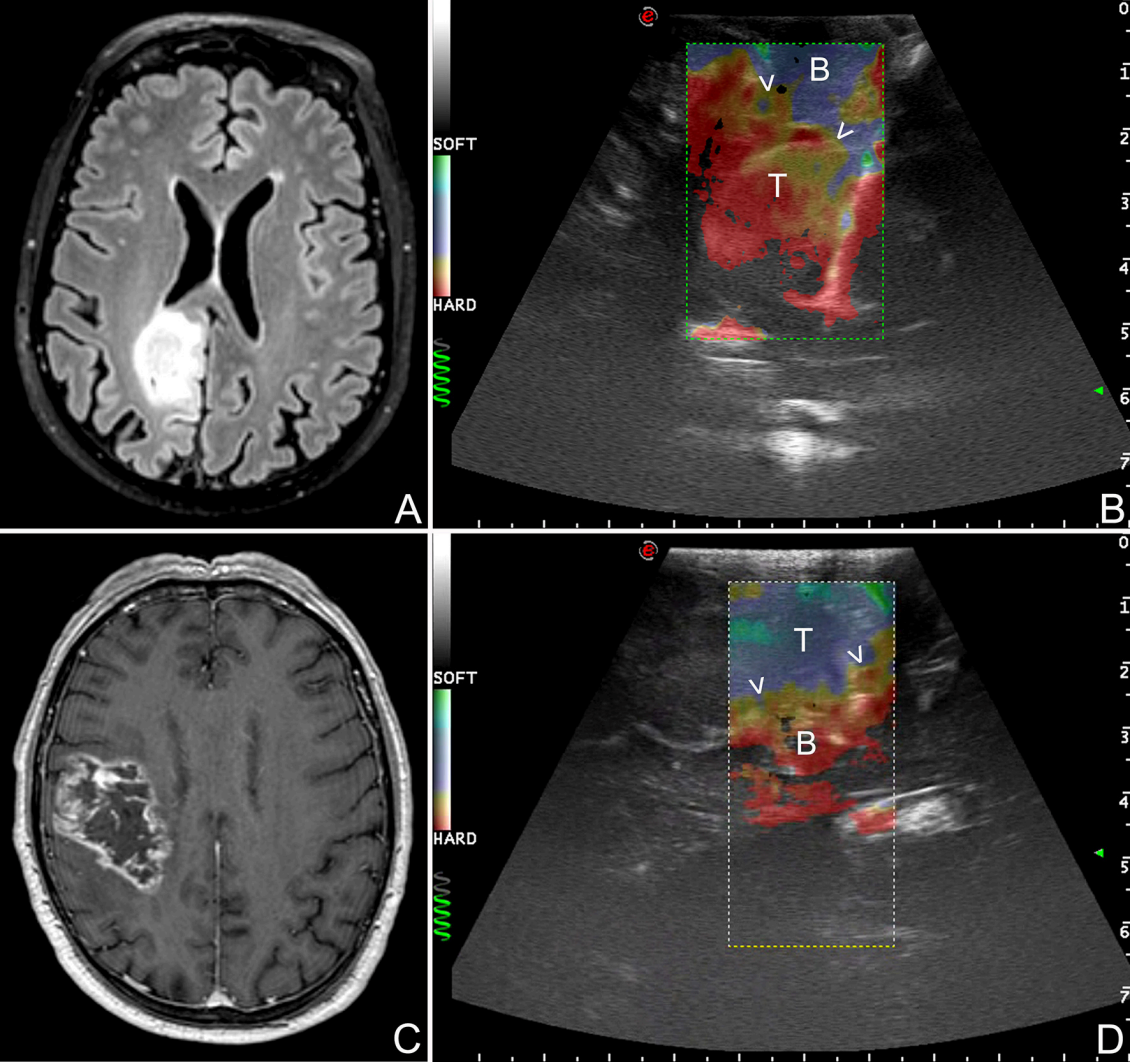
Strain elastography (SE) scans in different glioma grades. **(A,B)** SE in a case of right parietal low grade glioma. **(C,D)** SE in a case of right fronto-parietal glioblastoma. SE is able to differentiate between LGG and HGG relying on their stiffness. LGG appear stiffer than brain whereas HGG softer. Furthermore, SE aid in identifying tumor borders and in distinguishing tumor and edema. (T: tumor, B: brain, arrow heads: interface between tumor and surrounding brain).

Finger palpation has always been used in medicine. US elastography (ESG) represents the evolution of this approach. Applying a force to a tissue is possible to obtain a deformation that is related to its intrinsic mechanical characteristic, namely Young's E modulus (measure of the stiffness of a solid material) (70, 71). There are several techniques to measure and represent the elastic property of a tissue (70–72). Outside of the liver the most employed elastographic techniques are shear wave elastograhy (SWE) and strain elastography (SE). SWE belongs to dynamic elastosonography and involves a focused US stimulus to induce a micrometric displacement to obtain share waves, which propagate orthogonally in the tissue (72). SWE provides both quantitative and qualitative information on tissue stiffness. SE is a quasi-static elastographic modality based on a mechanical stimulus to induce a tissue deformation which is measured by a high-frequency serial US acquisitions (70–72) (Figures 4, 9). SE is more diffuse than SWE but again is capable only of qualitative measures.

Our group has focused the attention on SE demonstrating the feasibility in large-scale cohort of oncological-neurosurgery patients with the aims of lesions discrimination and characterization. Our SE exam is usually conducted before dura opening, maintaining the probe stationary and exploiting brain pulsatility as described by other Authors (73). In 64 patients we did not observed damage or adverse effect and we were able to discriminate lesion volume. In glioma subgroup we observed that in most of cases SE provide a lesion representation superimposable to standard B-mode but with a sharper margins visualization (Figures 4, 9). More importantly, SE demonstrated of being able to discriminate between LGG and HGG with a 85.7% of sensitivity and 94.7% of specificity (Figure 9). Indeed in most of cases LGG appear stiffer while HGG softer than surrounding brain parenchyma thus allowing to differentiate these tumor through an intra-operative US scan (Figure 9).

Our findings are aligned with the results obtained from other groups with SE and with SWE (10, 73–77). In any case, even if these results are really encouraging, elastosonography still must be considered an experimental technique.

## Conclusion

ioUS represents a pivotal adjunct to the existing surgical armamentarium, especially for a delicate application such as glioma surgery. ioUS is a polyvalent real-time imaging technique able to provide a great amount of information both anatomical and functional. Exploiting the advantages of each modality (B-mode, fusion imaging, Doppler-, CEUS, Elastography) it is possible to overcome several limitations of ioUS and to study glioma under various aspects. However, ioUS is an imaging technique that is rather demanding, requiring a specific training for each modality and in general for US semiotics, US physics and “knobology.”

In our opinion ioUS should be part of a multimodal comprehensive approach for surgical guidance in glioma resection also encompassing other imaging and functional modalities in a synergistic and complementary fashion.

## Author Contributions

MD, FP: study concept and design. FP, MD, and FD critical revision of the manuscript for intellectual content. FP, MD, CC, MS, FL, AP, LM, AS, IV, and FD acquisition of data, data analysis, and interpretation. FP, MD, and FD: study supervision.

### Conflict of Interest Statement

The authors declare that the research was conducted in the absence of any commercial or financial relationships that could be construed as a potential conflict of interest.
